# Evaluations of Electrostatic Filtration Efficiency and Antibacterial Efficacy of Antibacterial Electret Polypropylene Filters: Effects of Using Low Molecular Antibacterial Agent as Additive

**DOI:** 10.3390/polym13193303

**Published:** 2021-09-27

**Authors:** Jia-Horng Lin, Ying-Huei Shih, Chen-Hung Huang, Mei-Feng Lai, Shu-An Lee, Bing-Chiuan Shiu, Ching-Wen Lou

**Affiliations:** 1 College of Material and Chemical Engineering, Minjiang University, Fuzhou 350108, China; jhlin@fcu.edu.tw (J.-H.L.); toyysbk2@yahoo.com.tw (B.-C.S.); 2 Laboratory of Fiber Application and Manufacturing, Department of Fiber and Composite Materials, Feng Chia University, Taichung 40724, Taiwan; syhlhpc1990715@hotmail.com (Y.-H.S.); lai3630@gmail.com (M.-F.L.); 3 School of Chinese Medicine, China Medical University, Taichung 40402, Taiwan; 4 Advanced Medical Care and Protection Technology Research Center, College of Textile and Clothing, Qingdao University, Qingdao 266071, China; 5 Department of Aerospace and Systems Engineering, Feng Chia University, Taichung 40724, Taiwan; 6 Department of Environmental Engineering and Science, Feng Chia University, Taichung 40724, Taiwan; salee@fcu.edu.tw; 7 Department of Bioinformatics and Medical Engineering, Asia University, Taichung 41354, Taiwan; 8 Department of Medical Research, China Medical University Hospital, China Medical University, Taichung 40402, Taiwan; 9 Fujian Key Laboratory of Novel Functional Fibers and Materials, Minjiang University, Fuzhou 350108, China

**Keywords:** polypropylene, electret, triclosan, antibacterial, filter

## Abstract

In recent years, air filtration has been gaining much attention, and now people are much more concerned about antibacterial filters due to the spreading of COVID-19. The electret polypropylene (PP) nonwoven fabrics possess excellent filtration efficiency but a limited antibacterial effect against *S. aureus* and *E. coli*, and therefore triclosan is used in this study. Serving as an antibacterial agent, triclosan with a low molecular weight is an effective additive for the test results, indicating that the presence of triclosan strengthens the antibacterial effects of the filters. In addition, triclosan also strengthens the PP’s crystallinity, which in turn betters the filtration efficiency of the filters concurrently. Demonstrating powerful filtration and antibacterial performances, the antibacterial electret PP filters are highly qualified for filter applications.

## 1. Introduction

Air filtration has been a popular research topic because there are increasingly more studies substantiating that suspended particles do harm to the human body [1,2,3,4,5,6]. The majority of commercially available air filters applied to life are commonly made of electret filters. For example, masks, air purifiers, and air conditioner filters mostly consist of electret filters that exhibit highly efficient filtration and a low resistivity. Meanwhile, it is crucial that electret filters carry and preserve the electrostatic charges [7,8,9,10,11].

In many studies, different additives are used to improve the electret filter in terms of the loading capability of electrostatic charges [12]. For instance, Zhang et al. used boehmite nanoparticles to enhance PAN’s capability of carrying electrostatic charges, the efficiency of which reached 99.96%. The mechanism was that the incorporation of boehmite nanoparticles could provide PAN fibers with a greater number of deep energy traps, increasing the amount of electrostatic charges [13]. Moreover, Liu et al. tried to change the crystallinity and crystal morphology employing bicomponent filters and magnesium stearate with an attempt to enable electrostatic charges to enter deeper zones. As a result, the filters demonstrated a better filtration efficiency that was as high as 98.94% [14]. In addition, He et al. incorporated schorl powders, a natural electret filter, to strengthen the composite air purifiers. The proposed materials exhibited an excellent quality factor of filtering particles at sizes of 1–10 μm [15].

Due to the outbreak of COVID-19 in the last two years, there are increasing demands on antibacterial masks and air filters. The current masks perform antibacterial efficacy that depends on the electret materials; however, with the passing of preservation and service life, the dielectric materials gradually lose the electric charges which subsequent leads to an attenuated antibacterial efficacy. Besides, one research indicates that statistically, 7% of the population have a casual attitude toward the disposal of used masks [16], inflicting the environment with pollution and epidemic risk. Therefore, the reinforcement in antibacterial performance of air filters is considered a valuable perspective for studies. Xiao et al. added AgNPs to PVDF nanofiber membranes for the reinforcement in antibacterial efficacy, which was substantiated effectively resisting against S. aureus and E. coli. The bacterial reduction rate (BR) was 99.6%, the filtration efficiency was 99.95% and the pressure drop was 137.5 Pa [17]. Ungur et al. incorporated CuO with nanofiber membranes to improve the antibacterial efficacy, after which the microbes began to die by degrees [18].

Sun et al. compared two groups of air filters, one group contained electrical energy while the other group consisted of antibacterial agent. The antibacterial agent-contained filters could effectively reduce the bacterial survival rate to 10% in 100 min whereas the charged filters could only reduce the bacterial survival rate to 30% in 300 min [19]. Additionally, other studies selected different antibacterial agents for the filters, e.g., ZnO [20], silver nanofibers [21], and nano-TiO_2_ [22]. From the literature, there are few studies investigating how a combination of an antibacterial agent and polymers influences electret materials, for which triclosan that has a low molecular weight is used in this study. Triclosan is a commonly used broad-spectrum antimicrobial agent with good stability but without odor or smell, and therefore used in the toothpaste, mouthwash, and other fields [23,24]. Meanwhile, triclosan is proved to have a marginal sensitizing potential for eczema patients, and is skin allergy free when used in masks or filters [25]. To sum up, triclosan is perceived as a comparatively safer antibacterial agent. The meltability attribute of triclosan is utilized to facilitate the crystallinity of polypropylene (PP), after which the antibacterial efficacy and electrostatic charges of electret filters are evaluated to examine the effects of triclosan.

## 2. Materials and Methods

### 2.1. Materials

PP powders (Metocene MF650Y, Polymirae Co., Ltd., Seoul, Korea) are a homopolymer with a melt flow rate of 1800 g/10 min (230 °C, 2.16 kg). Triclosan (USP27, Great Chain Chemical Ltd., Taipei, Taiwan) is INCI 2,4,4-trichloro-2-hydroxyldiphenyl ether and has a melting point being 56–57.1 °C. NaCl (Shimakyu’s Pure Chemicals Co., Ltd., Samut Sakhon, Thailand) has a purity of 99.8%, serving as the particles used in this filteration test.

### 2.2. Preparation of Antibacterial Electret PP Filters

PP powders and triclosan (0.03, 0.06, or 0.09 wt%) are blended and processed with granulation. Via a melt-blown machine (Tianjin Shengruiyuan Machinery Technology, Tianjin, China), PP/triclosan pellets are processed into antibacterial PP nonwoven fabrics that are then processed with a corona charging device (Tai Ho Machinery Co., Taichung, Taiwan) at an ambient temperature of 25 °C and a relative humidity of 40%, during which the electric field is equipped with 1.5, 2.0, and 2.5 kV/cm. Different antibacterial electret PP filters serve as the experimental groups while the electret pure pp nonwoven fabrics are the control group. 

### 2.3. Surface Voltages and Surface Potential

A static meter (Model 5740, TAKK Industries Inc., Cleves, OH, USA) is used for the measurement. The measurement distance is 10 cm and the test results are recorded. The surface potential is computed according to the surface voltages.

(1) $$\mathrm{Surface}\;\mathrm{Potential}=\frac{V}{V_{0}}$$

where V_0_ is the initial surface voltage of antibacterial electret pp filters, and V is the residual surface voltage of antibacterial electret pp filters after they are stored in the standard environment for a certain length of time.

### 2.4. Filtration Efficiency and Pressure Drop

The filtration efficiency measurement is conducted with a gas flow being 85 ± 4 L/min and a particulate concentration being 200 mg/m^3^ using an electrical low-pressure impactor (ELPITM, Dekati, Finland). Samples are measured for the particle amount before and after they filter the air flow. The results are used to compute the filtration efficiency with the equation as follows.

(2) $$\mathrm{FE}=\frac{C_{0}-C_{i}}{C_{0}}\times 100\;\%$$

where FE means the filtration efficiency, C_0_ is the particle concentration before the filtration, and C_i_ is the particle concentration after the filtration.

Besides, a micromanometer (Models PVM 610, Airflow Measurements Ltd., Bolton, UK) is used to measure the pressure drop in front of and on the back of the filters during the filtration efficiency test. Afterwards, the quality factor is computed using the equation as follows.

(3) $$\mathrm{Quality}\;\mathrm{Factor}=\frac{-\ln\left(1-E\right)}{\Delta P}$$

where E is the filtration efficiency and ∆P is the pressure drop.

### 2.5. Differential Scanning Calorimeters

A differential scanning calorimeter (DSC, Q200, TA Instruments, New Castle, DE, USA) is used to measure the samples in terms of the enthalpy and crystals of fusion at increments of 10 °C/min. The crystallinity is computed using the equation as follows.

(4) $$\mathrm{Xc}\left(\%\right)=\frac{\Delta H_{f}}{\Delta H_{f}^{0}}\times 100\%$$

where X_c_ is the crystallinity, ∆H_f_ is the apparent enthalpy of crystallization, and $\Delta H_{f}^{0}$ means the enthalpy of crystallization when PP has a 100% crystallinity.

### 2.6. Inhibition Zone

Based on JIS L 1902:2008 qualitative test, samples are trimmed into a circular form with a diameter of 25 mm. *Escherichia coli* (*E. coli*) and *Staphylococcus aureus* (*S. aureus*) are used in this test. The bacterial suspension (0.1 mL) is smeared over a nutrient agar, and then covered by a sample. It is observed whether there is an inhibition zone surrounding the sample after the 24-h culture.

## 3. Results and Discussions

### 3.1. Surface Voltage and Surface Potential

**Figure 1:**
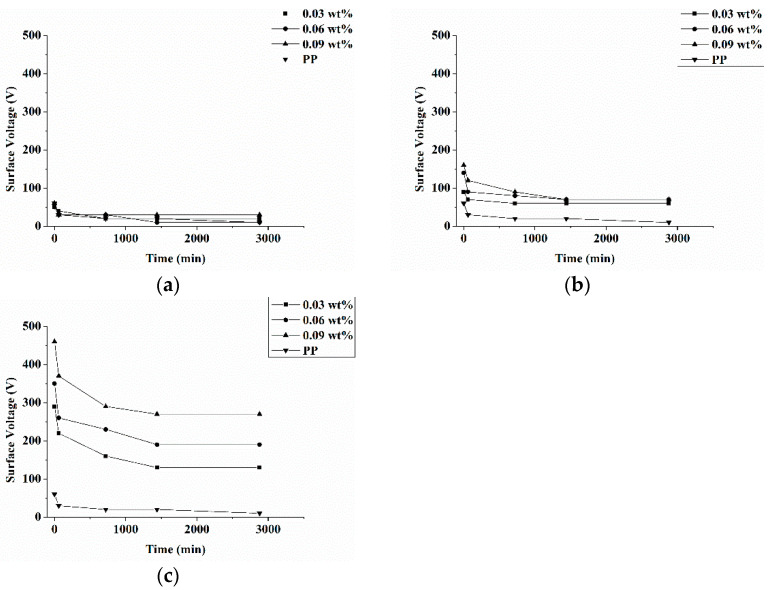
Surface voltage of antibacterial electret PP filters as related to triclosan amount (0.03, 0.06, and 0.09 wt%) and electric field intensity being (**a**) 1.5 kV/cm, (**b**) 2.0 kV/cm, and (**c**) 2.5 kV/cm.

Figure 1 shows that a rise in the electric field intensity has a positive influence over the surface voltage of antibacterial electret PP filters. The surface voltage significantly increases because of a higher electric field intensity as in Figure 1b,c. The higher the electric field intensity, the higher the energy density. As a result, antibacterial PP filters are enabled to keep more electric charges than the control group (i.e. electret pure PP nonwoven fabrics). A storage in an electric field intensity being 2.0 kV/m for a certain length of time (Figure 1b), all antibacterial electret PP filters demonstrate comparable surface voltage. However, with an electric field intensity being 2.5 kV/cm (Figure 1c), there are distinctive variations in the surface voltage. To sum up, 2.0 kV/cm is too low to load electric charges over antibacterial electret PP filters.

**Figure 2:**
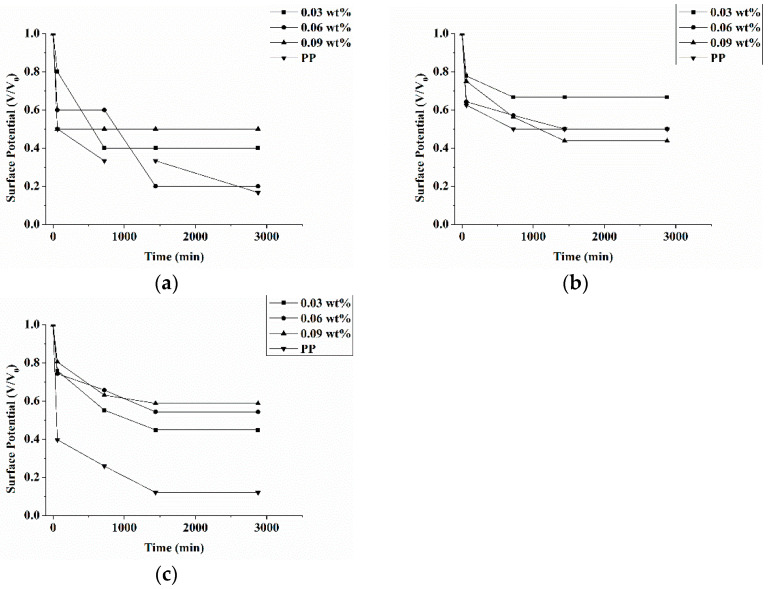
Surface potential of antibacterial electret PP filters as related to triclosan amount (0.03, 0.06, and 0.09 wt%) and electric field intensity at (**a**) 1.5 kV/cm, (**b**) 2.0 kV/cm, and (**c**) 2.5 kV/cm.

To substantiate how the triclosan amount is correlated with the preservation capacity of electric charges, equation (1) is used to compute the surface potential (Figure 2). In Figure 2a,b, there are no significant trends in the preservation capacity of electric charges because the corona charging device fails to provide enough electric energy when at 1.5 or 2.0 kV/cm. By contrast, Figure 2c shows that when the device supplies enough electric energy, antibacterial electret PP filters that contain 0.09 wt% of triclosan exhibit the optimal preservation capacity of electric charges. Moreover, it is also substantiated that the optimal surface voltage and surface potential occur when 0.09 wt% of triclosan is incorporated (Figure 1 and Figure 2). The amounts of triclosan lead to different levels of crystallinity.

Table 1 shows that the higher the triclosan amount, the better the crystallinity of antibacterial electret PP filters. That the surface voltage and surface potential are dependent on the crystallinity can be interpreted by the theory depicting that electric charge traps are presented in the crystalline region and amorphous region. There are variations in the electrical properties between the two regions, so the transmission of electric charge demands extra electric energy, which in turn allows the space between two regions to detain more electric charges. Therefore, this area is called a trap, and more traps can better preserve more electric charges [26,27]. To sum up, the incorporation of low molecular triclosan does not attenuate the surface voltage and surface potential, namely the antibacterial agent (i.e., triclosan) does not exert negative effects over the electret filters. Conversely, triclosan owns meltability, suggesting that triclosan is an ideal additive with a high potential for electret filters.

### 3.2. Inhibition Zone (Electret PP Nonwoven Fabrics)

**Figure 3:**
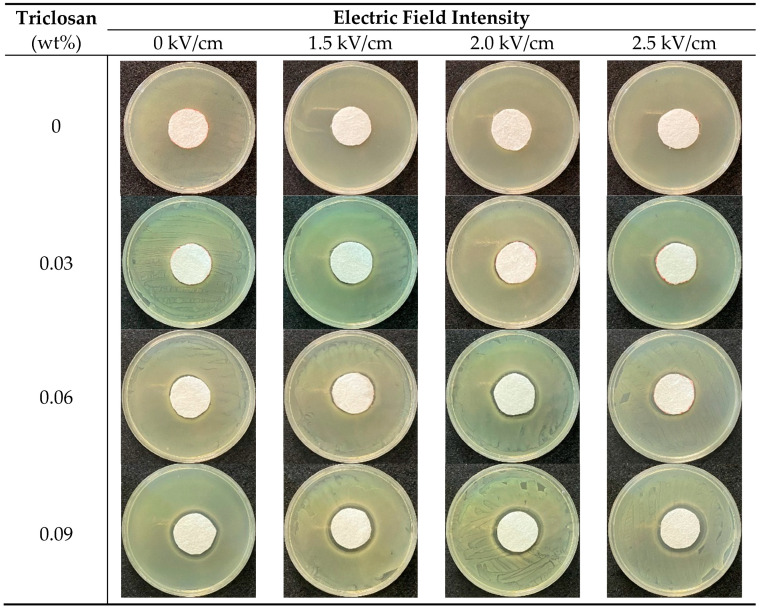
Inhibition zone against *E. coli* of samples as related to triclosan amount (rows 1–4: 0, 0.03, 0.06, and 0.09 wt%) and electric field intensity (columns 1–3: 1.5, 2.0, and 2.5 kV/cm).

**Figure 4:**
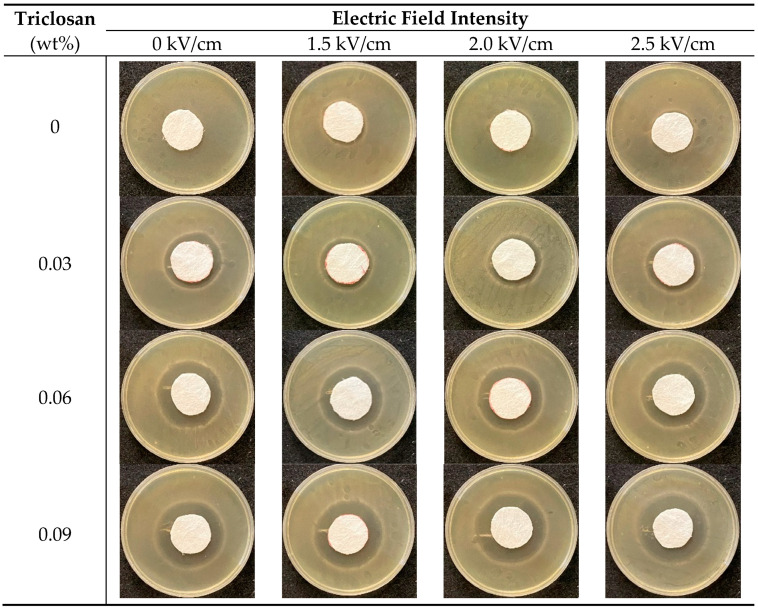
Inhibition zone against *S. aureus* as related to triclosan amount (rows 1–4: 0, 0.03, 0.06, and 0.09 wt%) and electric field intensity (columns 1–3: 1.5, 2.0, and 2.5 kV/cm).

Figure 3 and Figure 4 and Table 2 demonstrate that the control group, electret pure PP nonwoven fabrics containing 0 wt% of triclosan, possesses electric charges and thus exhibits antibacterial efficacy against *S. aureus,* but *E. coli* is a Gram-negative bacteria and has a thinner cell wall that contains phosphatide [28]. By contrast, *S. aureus* is a Gram-positive bacteria that does not contain phosphatide, and therefore electric charges demonstrate a more powerful antibacterial effect against *S. aureus*. Additionally, with a specified triclosan content, the inhibition zone is in direct proportion to the electric field intensity. A rise in the electric field intensity provides electret pure PP nonwoven fabrics with a greater surface voltage, which contributes to a higher antibacterial efficacy.

On the other hand, the antibacterial electret PP filters that contain 0.03 wt% of triclosan show antibacterial efficacy against both *E. coli* and *S. aureus*. Regardless of the bacteria type, the higher the triclosan amount, the better the antibacterial efficacy. Because triclosan constrains the synthesis of fatty acids, fatty acid is indispensable for bacteria, and triclosan reduces the microbes efficiently [29,30]. In Table 2, samples have a greater trend in inhibition zone when the triclosan content increases from 0.03 wt% to 0.06 wt% than when the triclosan content increases from 0.06 wt% to 0.09 wt%. This result is surmised due to the crystallinity of electret PP nonwoven fabrics. Table 1 shows that the crystallinity significantly increases when the triclosan content increases. Additionally, Kamalipour et al. indicated that the triclosan release rate was mitigated because of the increasing crystallinity [23]. As a result, when composed of 0.09% of triclosan, samples exhibit an inhibition zone that grows to a lower extent. In particular, the employment of both triclosan and electret treatment contributes a synergistic effect that provides the filters with an even greater antibacterial efficacy.

### 3.3. Filtration Efficiency, Pressure Drop and Quality Factor

**Figure 5:**
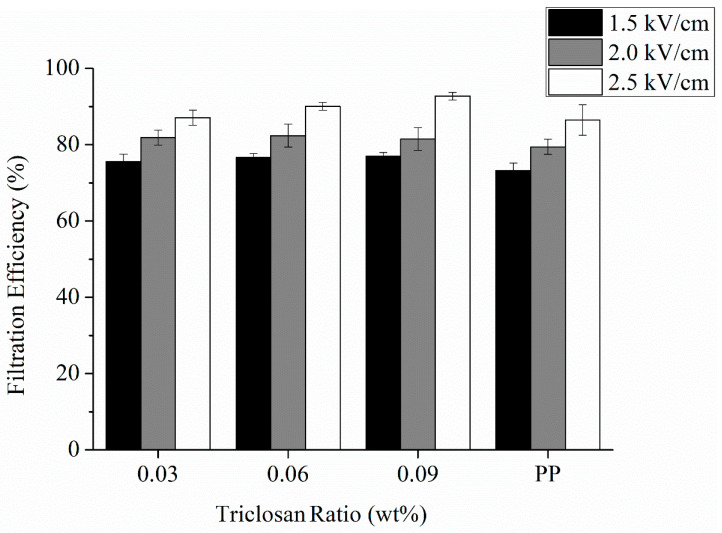
Filtration efficiency of antibacterial electret PP filters as related to triclosan amount (0.03, 0.06, and 0.09 wt%) and electric field intensity (1.5, 2.0, and 2.5 kV/cm). The control group (electret pure PP nonwoven fabrics) contains 0 wt% of triclosan.

The antibacterial electret PP filters demonstrate filtration efficiency that is associated with the amount of electric charges. Figure 5 shows that the results are consistent with the pattern that filtration efficiency is in direct proportions to the electric charge amount. Additionally, the trend shows that a rise in both of the electric field intensity and triclosan amount has a positive influence over filtration efficiency. The results can be interpreted by the previous discussion regarding the surface voltage. Electric charges play a role that adsorbs particles, which helps detain a greater number of particles at sizes smaller than the pore size of the filter. Hence, antibacterial electret PP filters achieve a better filtration efficiency.

**Figure 6:**
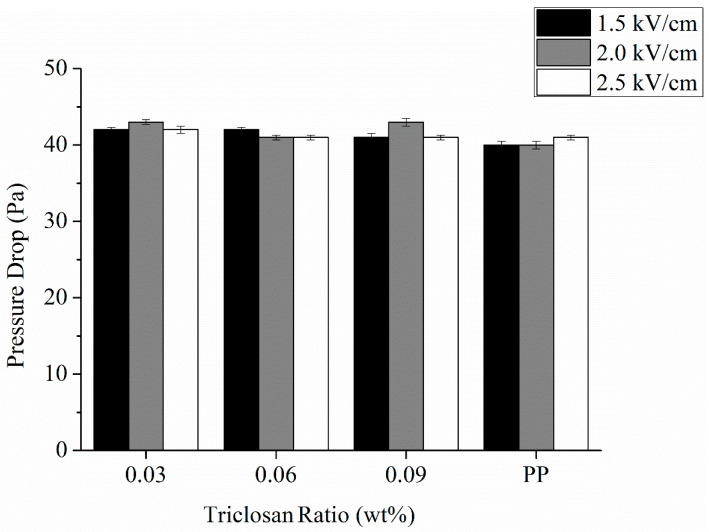
Pressure drop of antibacterial electret PP filters as related to triclosan amount (0.03, 0.06, and 0.09 wt%) and electric field intensity (1.5, 2.0, and 2.5 kV/cm). The control group (electret pure PP nonwoven fabrics) contains 0 wt% of triclosan.

**Figure 7:**
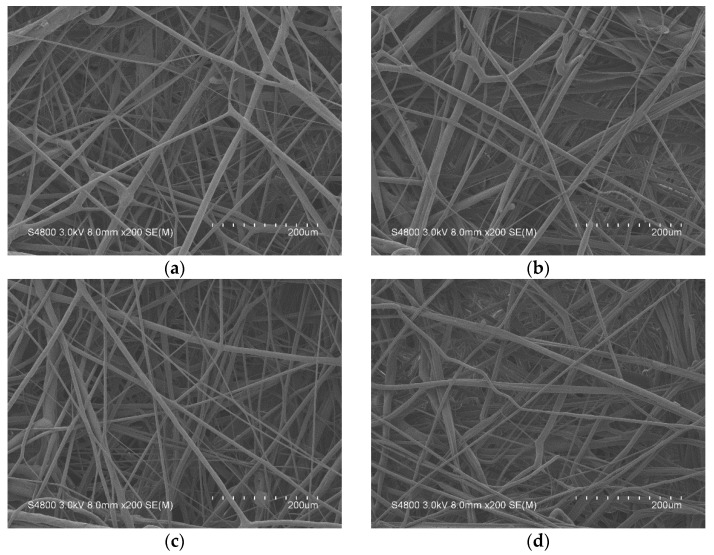
SEM images of antibacterial electret PP filters as related to the triclosan content being (**a**) 0 wt%, (**b**) 0.03%, (**c**) 0.06 wt%, and (**d**) 0.09 wt%.

Besides, the pressure drop also can serve as an important index for the air permeability, and a high pressure drop means a low air permeability. One advantage of antibacterial electret PP filters is that they have a strengthened filtration efficiency without a requirement to change the pressure drop. Figure 6 shows that all experimental groups (antibacterial electret PP filters) exhibit a pressure drop of approximately 40 Pa, which is attributed to the fact that antibacterial electret PP filters are composed of a comparable thickness and fabric structure as Table 3 and Figure 7.

**Figure 8:**
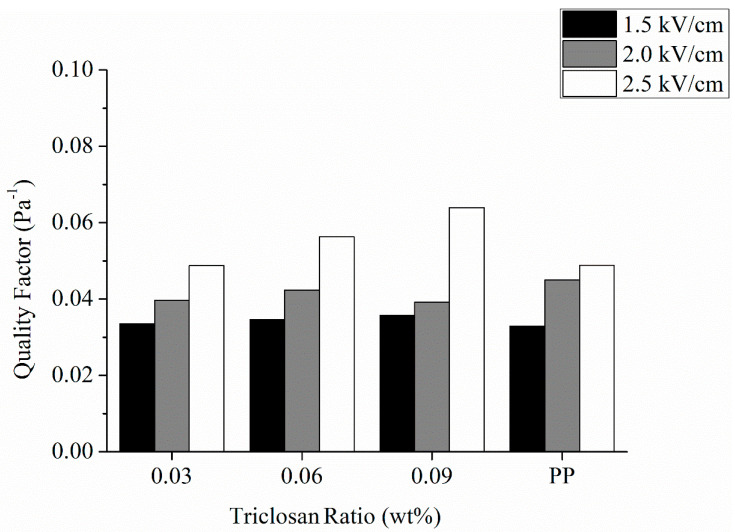
Quality factor of antibacterial electret PP filters as related to triclosan amount (0.03, 0.06, and 0.09 wt%) and electric field intensity (1.5, 2.0, and 2.5 kV/cm). The control group (electret pure PP nonwoven fabrics) contains 0 wt% of triclosan.

Generally speaking, it is not feasible to compare the filtration efficiency and pressure drop directly when the structure and thickness of filters are different. To solve this issue, the quality factor is then employed for the comparison. According to Figure 8, when there is a higher electric field intensity and a greater triclosan amount, there is a higher quality factor concurrently. When the pressure drop is specified, a higher efficient filtration means a greater quality factor equivalently.

## 4. Conclusions

In this study, the incorporation of triclosan has been substantiated as effective to improve the antibacterial efficacy of electret PP nonwoven fabrics. There is also a synergistic effect of the use of triclosan and electret treatment on the antibacterial performance of the filters. In addition, it takes only 0.09 wt% of triclosan to improve the crystallinity of PP to a considerable extent. In the meanwhile, the surface voltage is highly strengthened, so is a case for the filtration efficiency. In conclusion, the test results substantiate that triclosan, a low molecular antibacterial agent, is a qualified candidate as a powerful reinforcement for antibacterial electret filters.

## Figures and Tables

**Table 1:** Thermal properties of antibacterial electret PP filters as related to the triclosan amount being 0.03, 0.06, and 0.09 wt%.

Triclosan Ratio (wt%)	T_c_ (°C)	T_m_ (°C)	Crystallinity (%)
**0 (Pure PP)**	121.04	146.57	6.87
**0.03**	121.38	150.43	13.80
**0.06**	119.95	150.77	17.42
**0.09**	116.82	145.08	20.63

**Table 2:** Effects of triclosan amount (0, 0.03, 0.06, and 0.09 wt%) and electric field intensity (1.5, 2.0, and 2.5 kV/cm) on inhibition zone against *E. coli* and *S. aureus*.

Triclosan Ratio (wt%)	Electric Field Intensity (kV/cm)	*E. coli* Inhibition Zone (mm)	*S. aureus* Inhibition Zone (mm)
**0 (Pure PP)**	0	0	0
	1.5	0	0.7 ± 0.14
	2.0	0	0.8 ± 0.13
	2.5	0	0.9 ± 0.13
**0.03**	0	0.5 ± 0.10	2.0 ± 0.10
	1.5	0.8 ± 0.15	2.1 ± 0.08
	2.0	0.8 ± 0.09	2.1 ± 0.15
	2.5	1 ± 0.07	2.5 ± 0.31
**0.06**	0	1.2 ± 0.19	6.0 ± 0.54
	1.5	1.2 ± 0.35	6.8 ± 0.37
	2.0	1.4 ± 0.18	7.0 ± 0.03
	2.5	1.7 ± 0.39	7.7 ± 0.40
**0.09**	0	1.9 ± 0.34	8.6 ± 0.41
	1.5	2.0 ± 0.35	8.9 ± 0.38
	2.0	2.2 ± 0.29	9.2 ± 0.92
	2.5	2.5 ± 0.42	9.3 ± 0.50

**Table 3:** Effects of triclosan amount over the physical properties of antibacterial electret PP filters.

Triclosan Ratio (wt%)	Basis Weight (g/m^2^)	Thickness (mm)	Fiber Diameter (μm)	Air Permeability (cm^3^/s/cm^2^)
**0 (control group)**	70.3 ± 4.1	0.65 ± 0.02	6.14 ± 3.88	63.65 ± 4.17
**0.03**	65.4 ± 3.8	0.63 ± 0.04	6.50 ± 2.11	62.14 ± 4.09
**0.06**	59.1 ± 5.7	0.66 ± 0.01	6.31 ± 3.58	61.06 ± 4.61
**0.09**	61.8 ± 2.4	0.65 ± 0.02	6.46 ± 3.58	67.56 ± 4.75

## Data Availability

All data relevant to the study are included in the article.
